# Reproductive Seasonality, Estrous Cycle, Pregnancy, and the Recurrence of Postpartum Estrus Based on Long-Term Profiles of Fecal Sex Steroid Hormone Metabolites regarding Zoo-Housed Female Golden Takins (*Budorcas taxicolor bedfordi*)

**DOI:** 10.3390/ani14040571

**Published:** 2024-02-08

**Authors:** Tomoki Yoshida, Yuki Shimokawa, Makoto Ohta, Mayo Takayanagi, Satoshi Kusuda

**Affiliations:** 1 The United Graduate School of Agricultural Science, Gifu University, Yanagido, Gifu 501-1193, Japan; tomoki.yoshida0906@gmail.com; 2 Wildlife Conservation Center, Tokyo Zoological Park Society, Hodokubo, Hino, Tokyo 191-0042, Japan; 3 Yokohama Zoological Gardens ZOORASIA, Yokohama Greenery Foundation, Kamishirane-cho, Asahi-ku, Yokohama, Kanagawa 241-0001, Japan; 4 Zoo Biology Research Center, Laboratory of Animal Reproduction, Faculty of Applied Biological Sciences, Gifu University, Yanagido, Gifu 501-1193, Japan

**Keywords:** golden takin, fecal sex steroid hormone, progesterone, estrogen, sexual maturity, breeding season, estrous cycle, reproductive behavior, pregnancy, zoo

## Abstract

**Simple Summary:**

The golden takin (*Budorcas taxicolor bedfordi*) is an endangered animal. While information on this species’ reproductive physiology is essential for its conservation, endocrinological studies are limited. Monitoring fluctuations in fecal sex steroid hormones is known to be a useful means of understanding reproductive physiological states such as pregnancy and estrus. In this study, we measured the concentrations of sex steroid hormone metabolites excreted in the feces of female golden takins and used the long-term fluctuations to determine their endocrine ovarian activity. This study indicates that the monitoring of fecal sex steroid hormone concentrations can be used to understand estrus and pregnancy and predict delivery.

**Abstract:**

This study investigates the non-invasive monitoring of the endocrine ovarian activities of captive female golden takins (*Budorcas taxicolor bedfordi*) based on long-term fecal sex steroid hormone metabolite dynamics. Fecal progesterone (P_4_) metabolite dynamics were monitored in nine females for 0.5–15 years between 2004 and 2022. Fecal estradiol-17β (E_2_) and estrone (E_1_) metabolites were measured during certain estrous cycles, and fecal E_1_ metabolite concentrations were measured during all gestation periods. The breeding season of the captive animals was mainly between May and December, and they were polyestrous animals whose breeding season begins during the long-day period. The onset of the breeding season occurred slightly earlier as age increased. The mean age (±SD) at puberty based on fecal P_4_ metabolite dynamics was 4.1 ± 2.9 years. The first conception ages ranged from 2.3–10.2 years. The mean estrous cycle period (±SEM) was 25.4 ± 1.1 days, and mounting and mating occurred in periods of low fecal P_4_ metabolite levels during the breeding season. The mean gestation period (±SD) from the estimated mating date to the calving date was 253.9 ± 5.7 days, and the fecal P_4_ metabolite distribution during pregnancy was bimodal. Fecal estrone metabolite levels gradually increased 21 weeks before delivery, peaked during the week of delivery, and then markedly decreased in the first week after delivery. Estrus resumed in the first April–August period after delivery (mean ± SD; 103.5 ± 40.9 days) or in May of the year after delivery (421.0 ± 16.5 days). This study revealed that the estrous cycle and pregnancy of female golden takins can be determined by fecal progesterone metabolite dynamics and that fecal estrone metabolite dynamics increases toward parturition and are useful for predicting the date of delivery. This endocrinological information is important for planned breeding efforts for the golden takins.

## 1. Introduction

The takin (*Budorcas taxicolor*) is a large herbivore belonging to the bovidae family and is classified into four subspecies based on habitat and morphological characteristics: the golden takin (*B. t. bedfordi*), the Mishmi takin (*B. t. taxicolor*), the Sichuan takin (*B. t. tibetana*), and the Bhutan takin (*B. t. whitei*) [1]. Since takin populations as of 2008 have declined by at least 30% in the last three generations due to over-hunting and habitat loss [2], this species has been granted Category I in Chinese wild animals of national priority protection, classified as Vulnerable in IUCN Red List of Threatened Species [2], and listed in Appendix II in CITES [3].

The golden takin is found in the Qinling Mountains of Shaanxi Province, China, and its population in China was estimated to be 21,200 individuals in 1990, while the Forestry Bureau of Shaanxi Province reported that it ranged between 4418 and 5720 in 2001 [1]. Therefore, the reproductive conservation of the golden takin is an urgent issue. As of October 2023, there were 71 takins in 23 zoos worldwide [4], and this population is managed as a target species for the EAZA ex situ program in Europe [5]. In Japan, the first pair of golden takins was imported from China in 1990, followed by one female in 1992, one male in 1993, and two males and two females in 2011. There were 30 births from the first delivery in 1996 to December 2022, and the Japanese zoo-housed population is increasing. As of December 2022, twenty-two golden takins were kept in Japan.

A more detailed understanding of reproductive physiology is important for promoting reproductive conservation. The information on endocrinology, especially that obtained by monitoring sex steroid hormone levels, is important for the breeding of this species. Blood is commonly used to monitor sex steroid hormone levels, but blood sampling involves inflicting stress on the animal and the risk of injury for the sampler. In addition, anesthesia is required for blood sampling for this species and carries the risk of injury or death. Since sex steroid hormones in the blood are mainly excreted in the excrement, fecal steroid monitoring has been deemed a non-invasive tool for performing a detailed examination of the reproductive physiological statuses of many wild mammals [6]. However, difficulties are associated with tracking specific individuals and regularly collecting the feces of wild golden takins due to their seasonal migration from 1360 m to 2890 m; they migrate to a high altitude in the summer (June–August), a low altitude in the spring (April–May) and autumn (September–November), and an intermediate altitude in the winter (December–March) [7]. Therefore, limited information is currently available on field reproduction. Although the sexual maturity, breeding season, gestation period, and season of birth of golden takins have been reported [8], their reproductive endocrinological characteristics remain unknown. In Sichuan takins, a takin subspecies, the fecal progesterone (P_4_) profile is important for clarifying estrous cycles and pregnancy [9], and fecal cortisol concentrations peak on the first day of delivery in some cases [10]. In male Sichuan takins, fecal testosterone and cortisol concentrations peak between May and August and between April and June, respectively [9,10].

The aim of this study was to identify basic reproductive characteristics related to sexual maturity, reproductive seasonality, the estrous cycle, gestation period, and the return of estrus using continuous sex steroid hormone profiles from the feces of captive female golden takins.

## 2. Materials and Methods

The animals used in this study were 9 female golden takins: Nos. 1–6 kept at the Tama Zoological Park (TZP; Hino, Tokyo, Japan; 35.65° N, 139.40° E) and Nos. 7–9 kept at Yokohama Zoological Gardens (YZG; Yokohama, Kanagawa, Japan; 35.50° N, 139.52° E) (Table 1). Both zoos are members of the Japan Association of Zoos and Aquariums and comply with the Animal Welfare Regulations and Standards of this association. This study was conducted in compliance with the Regulations for the Handling of Animal Experiments of Gifu University, and this university approved our study (No. 2022-071). All individuals were kept in natural conditions at each facility. Nos. 2–5 and Nos. 7–9 were kept in cohabitation with adult males during this study period. Females were cohabited with adult males during the day. However, No. 9 was cohabited with adult males at night from 24 to 25 September 2019. Also, No. 8 was cohabited with adult males in 2-day intervals from 8 February to 21 April 2019, and No. 9 was cohabited with adult males in 3-day intervals from 17 April to 18 May 2019. We collected 54, 1100, 1475, 1544, 556, 37, 578, 711, and 362 samples from Nos. 1–9, respectively. Feces were collected approximately 2 times weekly. However, there were times when collection was conducted once a week, such as before sexual maturity, during the pregnancy period, or when labor saving was necessary. On the other hand, No. 2’s feces were collected daily between 23 February 2005 and 30 March 2005 before and after birth. Fecal samples were stored at −20 °C in sealed plastic bags until analysis. During the period of cohabitation between No. 2 and the corresponding male in 2004–2007, the behavior of both sexes was recorded, and the presence or absence of the following behaviors was noted according to the 2012 report by Adkin et al. [9]: the flehmen response, sniffing genitals, mounting, or mating behavior exhibited by the male toward the female. The hours of light and dark per day were calculated using the time of sunrise and sunset in Tokyo and Yokohama from the National Astronomical Observatory of Japan’s calendar homepage [11] (https://eco.mtk.nao.ac.jp/koyomi/dni/ (accessed on 18 September 2023)).

Three grains of frozen feces were dried at 100 °C for approximately 6–8 h, and fecal steroids were extracted as previously reported [12]. Briefly, 0.1 g of fecal powder was extracted with 5 mL of 80% methanol in ultrapure water (*v*/*v*) and vortex-mixed for 30 min. After being left to stand at 4 °C overnight, the extraction solvent was centrifuged at 3000 rpm for 10 min, and the supernatant was diluted at a ratio of 1:10–200 using assay buffer (0.04 M of disodium hydrogen phosphate dihydrate buffer containing 0.1% bovine serum albumin in 0.15 M of sodium chloride).

Fecal concentrations of progestagen and estrogen were measured using enzyme immunoassays (EIAs), as previously reported [12,13]. The antibodies used were as follows: LC-28 (Aska Pharma Medical, Minato-ku, Tokyo, Japan), FKA302-E (Cosmo Bio, Tokyo, Japan), or AF17091287-001 (consigning production with Cosmo Bio, Tokyo, Japan) for progesterone (P_4_), FKA236-E (Cosmo Bio, Tokyo, Japan) for estradiol-17β (E_2_), and FKA234-E (Cosmo Bio, Tokyo, Japan) for estrone (E_1_). The main cross-reactivities of each antibody, except for AF17091287-001, were previously reported [12,13]. The horseradish peroxidases used were FKA301 (Cosmo Bio, Tokyo, Japan) for P_4_, FKA235 (Cosmo Bio, Tokyo, Japan) for E_2_, and FKA233 (Cosmo Bio, Tokyo, Japan) for E_1_. The cross-reactivities of the AF17091287-001 antibody were not provided by the manufacturer. We purchased each standard from Wako Inc. (Osaka, Japan), FUJIFILM Wako Inc. (Osaka, Japan), Sigma-Aldrich Japan (Tokyo, Japan), and Steraloids Inc. (Newport, RI, USA), and the examined cross-reactivities were 100% for P_4_ (161-14531), 14.85% for 5α-pregnane-3,20-dione (P7754), 2.95% for 4-pregnen-20α-ol-3-one (Q3600-000), and less than 1% for 4-pregnen-17α-ol-3,20-dione (H5752), testosterone (208-08341), androstenedione (A9630), estradiol-17β (052-04041), estriol (056-05301), estrone (E9750), corticosterone (086-02484), and cortisol (037-17583). Since these hormones are excreted in the feces as metabolites, we contend that this study actually captured metabolites that cross-reacted with the hormone antibodies, but for convenience, we used the hormone names of the antibodies used.

All sex steroid hormone concentrations in feces were expressed as content per one gram of dried feces. We initially examined the estrous cycle based on whether fecal P_4_ profiles showed a cyclic pattern. The estrous cycle is generally calculated using the above base lines. Baseline is defined as the mean + 1.5 to 2 standard deviations (SD) at the time when there is no more elevation after repeating this operation, excluding as elevation values greater than the mean + 1.5 to 2 standard deviations (SD) for all samples. However, in this method, we considered values above about 87~95% of the values of the total sample to be elevated. In cases such as the golden takin, for which the cycle continues for a long period of time, this method could not calculate progesterone elevation well. In case of No. 5, the increase period due to pregnancy or estrus cycle was longer than the low period during the non-breeding season, so the aforementioned calculation was not possible. In the case of the Asian elephant (*Elephas maximus*), an annual breeding animal whose progesterone levels fluctuate periodically, and in that of the white rhinoceros (*Ceratotherium simum simum*), which is known to have two patterns regarding estrous cycle length, it has been reported that the estrous cycles were calculated above a certain level [14,15]. Estrogen levels were measured during the estrous cycle of the golden takins, but their variation was not clear, and they were often not elevated when fecal progesterone metabolite levels were low. Therefore, based on the overall progesterone metabolite dynamics, it was decided to calculate the estrous cycles based on the fact that these levels are almost always less than 4 μg/g during the non-breeding season and continuously exceed 4 μg/g only during the estrous cycle. The estrous cycle, consisting of the gluteal and follicular phases, was calculated based on the P_4_ profile during the periods when feces were collected at least twice a week: (1) the luteal phase was defined as P_4_ concentrations > 4 μg/g on at least 2 consecutive points, and was calculated from the middle date of the pre- and post-points > 4 μg/g; (2) the follicular phase was calculated from the end date of the luteal phase to the start date of the next luteal phase; (3) the estrous cycle was calculated from the beginning date of the follicular phase to the end date of the luteal phase; (4) when points were not <4 μg/g and the next cycle had started, the date with the lowest value during the cycle was set as the end date of the estrous cycle, and these cycles were not used to calculate the luteal and follicular phases. The gestation period was determined to span from 5 days before the start date of the luteal phase to the delivery date based on the number of days from the final mating date (3 cases) to the point at which fecal P_4_ concentrations were >4 μg/g being 5 days. The start date of the breeding season was defined as the start date of pregnancy or the date before the average days of the follicular phase for each individual from the start date of the first luteal phase. The end date of the breeding season was the end date of the last estrous cycle. During the collection of feces conducted once a week, the breeding season was also defined as the period when P_4_ concentrations were > 4 μg/g on at least 2 consecutive points, and the beginning and end dates of the breeding season were counted. An increase of more than 4 μg/g at only one point was not considered to be indicative of the breeding season.

Average P_4_ and E_1_ concentrations at weekly intervals for 15 pregnancies were calculated from week 0 to the estimated mating date or the study-starting date for each pregnancy, with the period from 3 days before delivery to 3 days after delivery serving as week 0.

The pregnancy period spanned from the estimated mating date to the delivery date, the estrous cycle period spanned from the beginning of the first follicular phase to the end of the last luteal phase, and the non-estrus period consisted of the remaining time, the number of breeding seasons that started and ended is shown, and the number of births is counted by month. The beginning of the first follicular phase of each breeding season was taken as the start of the breeding season, and the end of the last luteal phase was taken as the end of the breeding season. The year of pregnancy was not taken to be the end of the breeding season.

## 3. Results

### 3.1. Sexual Maturity

**Figure 1:**
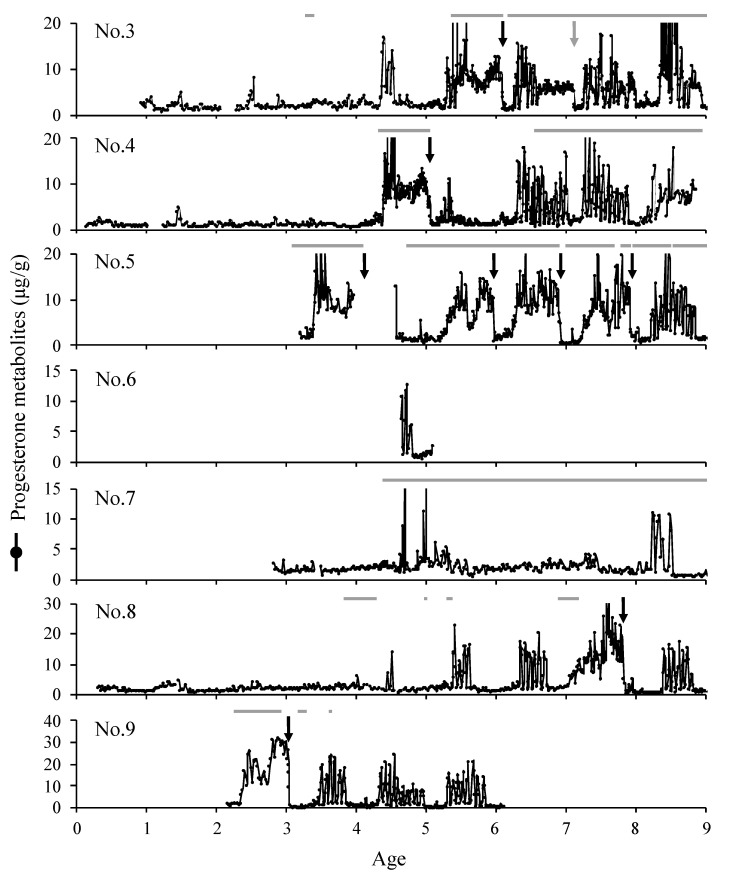
Fecal progesterone profiles of 7 female golden takins while aging. The black arrows and lone gray arrow indicate deliveries and miscarriage, respectively. Gray lines indicate periods of cohabitation with adult males.

Feces collection for nos. 3, 4, and 8 started when the animals were approximately one year old, and they had their first estrous cycles at 1.4, 1.4, and 5.4 years, respectively (Figure 1). No. 6 had already started its estrus cycle by the age of 4.7 years at the start of this study, and feces collection for No. 7 was started at the age of 2.8 years, while the first estrous cycle was observed at 8.2 years. Moreover, Nos. 5 and 9 became pregnant in the first estrus cycle of the year in which feces collection started, with ages at conception of 3.4 and 2.3, respectively. Nos. 3, 4, 5, 7, 8, and 9 had their first pregnancies during this study, and their ages at the start of their pregnancies were 5.4, 4.4, 3.4, 10.2, 7.1, and 2.3, respectively.

### 3.2. Breeding Season

**Figure 2:**
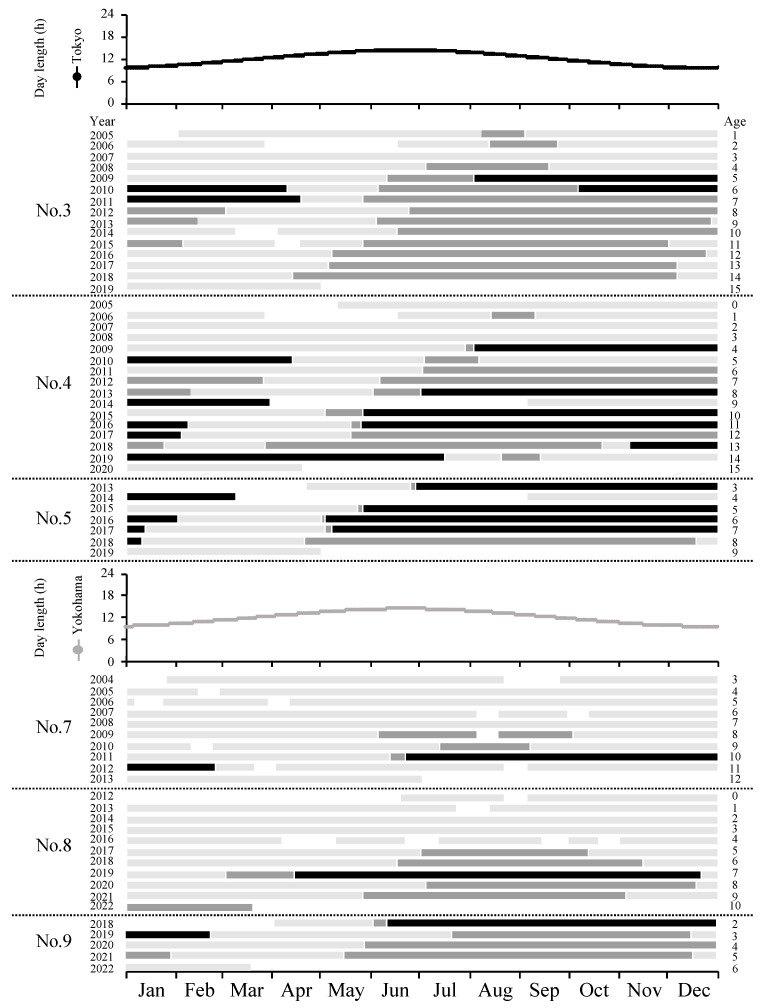
Transitions of non-estrus, estrous cycle, and pregnancy in 6 female golden takins during the entire study period. ■: non-estrus; ■: estrous cycle; ■: pregnancy. Day length correspond to Tokyo and Yokohama.

**Figure 3:**
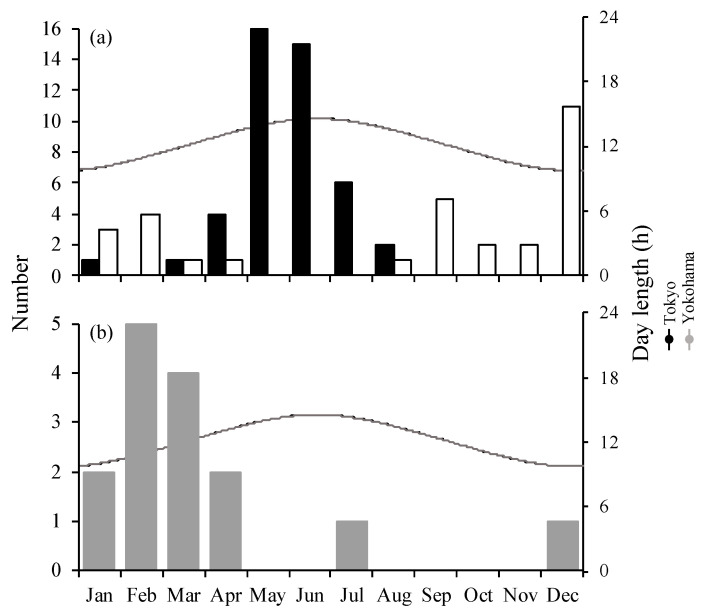
Day length and monthly total numbers for (**a**) the beginning (black bar) and end (white bar) of the breeding season and (**b**) delivery (gray bar) regarding female golden takins. Day length corresponds to Tokyo and Yokohama.

The onset of the breeding season for seven females was between January and August (*n* = 48), when the light period was longer (Figure 2), with the beginning of the breeding season occurring 16 times in May, 15 times in June, 8 times in July, and 4 times in August (Figure 3a). The end of the breeding season (i.e., the end of the estrous cycle), excluding pregnancies, was observed between August and March (*n* = 34), and 32% of all breeding seasons ended in December (Figure 3a). The onset of the breeding season for Nos. 3, 4, 5, 8, and 9 was slightly earlier as age increased (Figure 2). Additionally, in the absence of pregnancy, the average number of estrous cycles per breeding season (±SEM) was 6.6 ± 1.0 cycles (2.0–11.0 cycles) (Figure 2).

### 3.3. The Estrous Cycle and Reproductive Behavior

**Figure 4:**
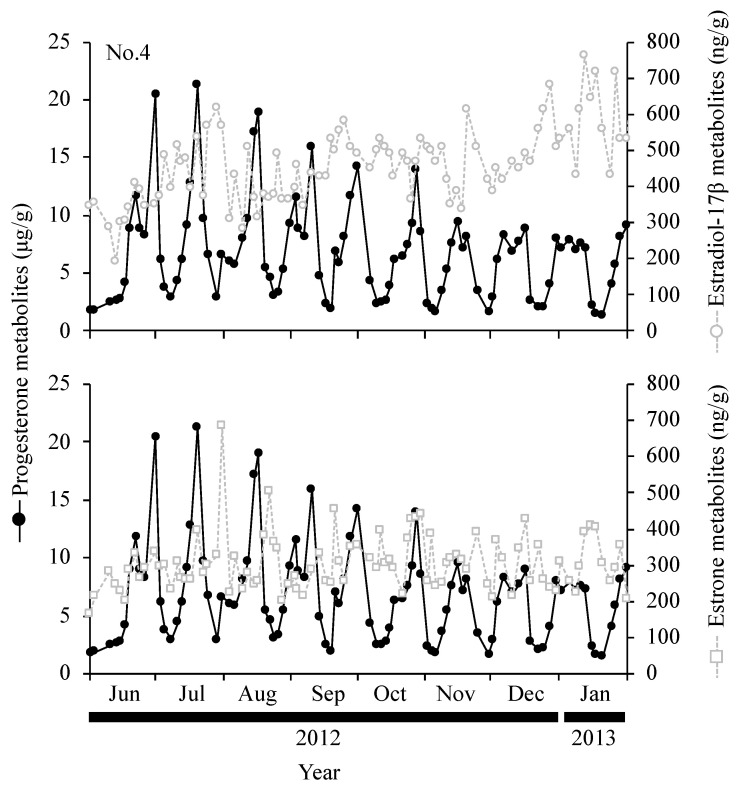
Fecal progesterone, estradiol-17β, and estrone profiles during estrous cycles for female golden takin No. 4.

**Figure 5:**
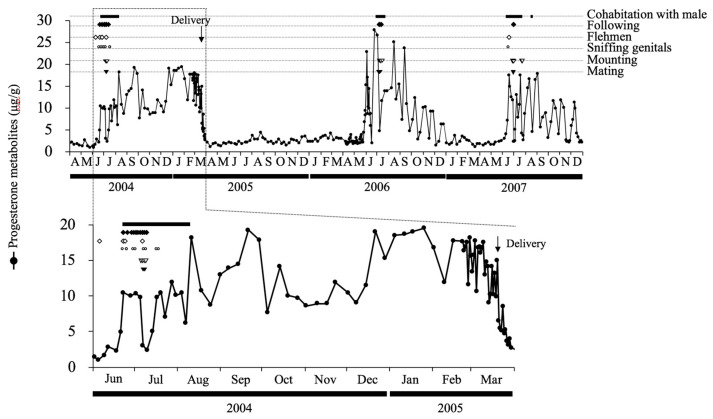
Fecal progesterone profile of female golden takin No. 2, and reproductive behaviors and day length. Black bar: cohabitation with male; ◆: following; ◇: flehmen; ○: sniffing genitals; ▽: mounting from male; ▼: copulation. Day length corresponds to Tokyo.

Estrous cycles were counted 150 times for seven females, with a mean estrous cycle length (±SEM) of 25.4 ± 1.1 days (21.8–31.6 days), a mean luteal phase length (±SEM) of 17.3 ± 1.1 days (15.2–24.0 days), and a mean follicular phase length (±SEM) of 8.5 ± 0.5 days (5.9–11.0 days). Fecal E_2_ and E_1_ concentrations also increased and decreased when there were cyclic variations in fecal P_4_; however, the dynamics of E_2_ and E_1_ did not overlap (Figure 4). Fecal E_2_ concentrations slightly decreased as fecal P_4_ concentrations increased, while fecal E_1_ concentrations slightly increased as fecal P_4_ concentrations decreased (Figure 4). Mounting and mating were observed during periods of low P_4_ concentrations (Figure 5). Flehmen was often performed before mounting and mating (Figure 5). Sniffing genitals and following were noted during cohabitation with males, regardless of fecal P_4_ profiles (Figure 5).

### 3.4. Pregnancy and Recurrence of Postpartum Estrus

Deliveries occurred 15 times for eight females during the study period (Figure 1). The number of deliveries was higher between January and April than between July and December, with five occurring in February and four occurring in March (Figure 3b). The mean duration of pregnancy (±SD) was 253.9 ± 5.7 days (247.0–270.5 days, *n* = 14).

**Figure 6:**
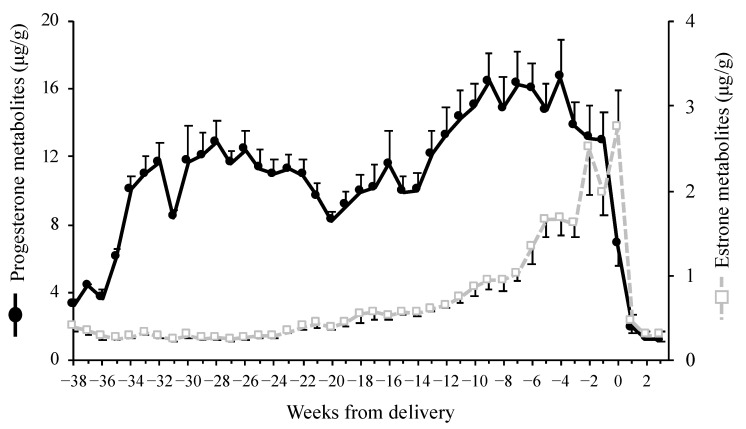
Mean weekly values (±SEM) of fecal progesterone and estrone concentrations for 13 pregnancies for 7 golden takins. The date of delivery was set as the middle of week 0.

Weekly P_4_ concentrations during pregnancy rapidly increased starting from 36 to 34 weeks before delivery, remained high starting from 34 to 22 weeks before delivery, and decreased slowly 22 to 20 weeks before delivery (Figure 6). Thereafter, they gradually increased starting from 20 to 4 weeks before delivery, at which point they peaked. Weekly P_4_ concentrations then decreased gradually 4 weeks to 1 week before delivery and declined sharply by the week after delivery. Weekly E_1_ concentrations during pregnancy increased gradually starting from approximately 21 weeks before delivery, peaked in the week of delivery, and then decreased sharply in the week following delivery (Figure 6).

Estrous regression was confirmed according to fecal P_4_ dynamics in 13 postpartum cases. Of these, one case in December was confirmed on day 197, and there were two cases in January on days 101 and 112, four cases in February on days 90 to 149, three cases in March on days 401 to 441, and two cases in April on days 57 and 83, and there was one in July on day 36.

The estrous regression date after delivery was between April and August after delivery or in May of the year after delivery, with means (±SD) of 103.5 ± 40.9 days (days 36–197, *n* = 10) and 421.0 ± 16.5 days (days 401–441, *n* = 3), respectively. Feces collection for No. 1, which gave birth in March 2004, and No. 7, which gave birth in February 2012, finished 328 and 494 days after delivery, respectively, and estrus did not return by the end date of sampling. No. 4, which gave birth in April 2010 and in February 2016, fed its calves via natural suckling and resumed estrus 83 and 104 days after delivery, respectively (Figure 1). On the other hand, No. 3 gave birth in April 2010, and No. 5 gave birth in February 2016, and their calves died on days 15 and 2 after delivery, respectively. Estrus resumed on days 57 and 90, respectively. One miscarriage occurred during the study period in April, and estrus resumed 39 days later (Figure 1).

## 4. Discussion

The fecal sex steroid hormone profiles of captive female golden takins examined for approximately 18 years provided information on their sexual maturity, breeding seasons, estrous cycles, gestation periods, and estrous regression. The average age at first conception in the present study was 5.5 years. Sexual maturity in the wild is 4–5 years [8], which is consistent with the present results. Moreover, some individuals started their first estrous cycle and first conception markedly earlier than wild individuals, with the first estrous cycle occurring at 1.4 years and the first conception occurring at 2.3 years. The ages of puberty and sexual maturity are affected by nutritional status, and large individual differences have been reported, even within the same species [16]. In addition, feeding with a diet with low energy or protein content delayed the onset of puberty in sheep (*Ovis aries*) [17]. Therefore, some individuals may have reached sexual maturity earlier in captivity due to the food-rich environment and individual differences.

The breeding season of female golden takins is between May and December, during which time days are long, and the golden takin is a polyestrous animal that starts its breeding season during the long-day period. The breeding season of wild golden takins is generally reported to be between June and October [8]. We found that the breeding season of captive Japanese individuals was approximately 3 months longer than that of wild individuals and that some individuals started 1–3 months earlier and ended 1–6 months later than wild individuals. In Japanese serow (*Capricornis crispus*), for which the breeding season in the wild is between September and January, the mounting of captive individuals was observed between November and April, and the breeding season was extended because these animals do not become pregnant in captivity [18]. Therefore, the extended end of the breeding season for captive golden takins may also be due to a lack of pregnancy. On the other hand, female Sichuan takins at the Lincoln Park Zoo (41.84° N, 87.68° W) exhibited cycles of fecal P_4_ concentrations between June and December [9], and this result is consistent with the breeding season observed in the present study. Wild golden takins migrate to an altitude of 1773 m in April–May, 2672 m in June–August, 1821 m in September–November, and 2115 m in December–March through the Qinling Mountains in accordance with plant phenology [7,19]. Captive golden takins may not follow their original breeding season due to the elimination of altitudinal movement and food restrictions. Additionally, for individuals whose monitoring started at less than 3 years of age, the onset of the breeding season was slightly earlier as age increased. For captive Japanese serows, the onset of the breeding season was also shown to occur earlier as age increased [20]. Therefore, the earlier onset of the breeding season for captive individuals than for wild individuals may be associated with the inherent potential of the takin brought about by the abundant food resources, freeing them from the limitations of altitudinal movement.

Fecal P_4_ profiles showed a marked increase or decrease seasonally in female golden takins, and this was considered to correspond to an estrous cycle. The average estrous cycle length was 25.4 days. The estrous cycle length of the golden takin was shown to be shorter than that of the Sichuan takin, whose average was 34.4 days [9]. However, the estrous cycle length in the present study varied by approximately 10 days depending on the individual and ranged between 25.7 and 49.0 days among Sichuan takins [9]. Among white rhinoceros, estrous cycles have been reported to range from 25 to 90 days [21], both of which are considered normal estrous cycles. Therefore, we hypothesized that the estrus cycle of the golden takin is normal, although there is large individual variation in the estrus cycle. No correlation was observed between estrous cycle length and age, and estrous cycles longer than 30 days were observed for many individuals (Nos. 2, 3, 5, 8, and 9). It currently remains unclear why estrous cycle lengths exceed 30 days and if these are normal estrous cycles for takins.

The present study is the first to report the estrogen profiles of takins. The results obtained show that fecal E_2_ concentrations slightly decreased as fecal P_4_ concentrations increased, while fecal E_1_ concentrations increased as fecal P_4_ concentrations decreased. Estrogen in the estrous cycle generally increases and decreases with follicular growth and surges with the LH surge. In muskoxen (*Ovibos moschatus*), which are classified under the same subfamily Caprinae like the golden takin, blood E_2_ concentrations decreased in the mid- and late luteal phase, blood LH concentrations peaked 2–5 days after progesterone had reached its baseline, and E_2_ concentrations increased on days 3–4 of estrus [22]. Decreases in E_2_ concentrations and increases in E_1_ concentrations during the estrous cycle in golden takins may be associated with follicular growth and LH surges. However, the changes observed were not distinct dynamics. Estrogen is mainly excreted in urine or feces [23]; ponies (*Equus caballus*), pigs (*Sus scrofa domesticus*), African elephants (*Loxodonta africana*), and Sumatran rhinos (*Dicerorhinus sumatrensis*) mainly excrete estrogen in urine, while sheep and white rhinos (*Ceratotherium simum*) mainly excrete it in feces [24,25,26,27].

In the same subfamily (Caprinae) as golden takins, chamois (*Rupicapra rupicapra*) and aoudad (*Ammotragus lervia*) showed non-periodic fecal estrogen profiles during the estrous cycle [12,28], while muskoxen exhibited increased blood estrogen levels in the follicular phase [22]. Furthermore, in goral (*Naemorhedus griseus*), fecal estrone glucuronide metabolite concentrations increased for 2–3 days in the follicular phase, and 64.9% of estrogen metabolites in feces were identified as estradiol [29]. In Okapi (*Okapi johnstoni*), which belong to the Giraffidae family, fecal estrogen dynamics examined using estradiol-17β antibodies did not show a clear pattern related to the estrous cycle [13], and estrogen dynamics in urine assessed using estrone-3-sulfate antibodies were reported to reflect the fluctuation associated with follicle growth [30]. Based on these findings, the estrogen dynamics during the estrous cycle of golden takins warrant further investigation, along with the measurement of urinary estrogen and the identification of fecal sex steroid hormone metabolites to which estrogen antibodies bind.

Mounting and mating occurred during periods of low fecal P_4_ concentrations in the breeding season, and male flehmen was observed before these behaviors. Sniffing genitals and following were noted during cohabitation with males, regardless of fecal P_4_ profiles. Among Sichuan takins, these behaviors, except for following, are defined as reproductive behaviors [9], and male flehmen is an external predictor of mating with and mounting a female.

In golden takins, fecal P_4_ concentrations remained high during pregnancy; fecal E_1_ concentrations gradually increased approximately 21 weeks before delivery; and the monitoring of fecal P_4_ and E_1_ concentrations is useful for a pregnancy diagnosis and predicting the date of delivery, respectively. Moreover, their fecal P_4_ profile during pregnancy showed a bimodal pattern, with E_1_ concentrations beginning to increase as the first peak in P_4_ concentrations decreased. Blood P_4_ concentrations in goats (*Capra*) [31] and sheep [32], the gestation periods of which last approximately 5 months, remained high during pregnancy. Additionally, plasma P_4_ concentrations in sheep increased rapidly starting from approximately 80 days of gestation [32], similar to the fecal P_4_ profiles during the pregnancy of golden takins. In goats, the main source of P_4_ secretion during pregnancy is the gestational corpus luteum [33], whereas it is gradually transferred from the gestational corpus luteum to the placenta in sheep [34]. In Sichuan takins, fecal P_4_ metabolite concentrations during the last third of pregnancy were 3-fold higher than those in the luteal phase [9]. The bimodal pattern of fecal P_4_ concentrations and the onset of increases in E_1_ concentrations may reflect placental growth and the transition of the main source of P_4_ secretion in golden takins.

The pregnancy period from the estimated mating date to the delivery date was 253.9 days, and deliveries mainly occurred between January and April. Wild golden takins give birth between early February and March after a pregnancy period of 8 to 9 months [8]. Furthermore, the pregnancy period of Sichuan takins in a North America zoo was 245 days, and the birth season was mainly between February and June [9]. The pregnancy period and birth season of zoo-housed golden takins examined in the present study were consistent with those of wild golden takins and zoo-housed Sichuan takins.

The estrous cycle resumed in the first April–August period after delivery or in May of the year after delivery, indicating that the breeding season resumed within two years at the latest for golden takins and that most individuals resumed breeding within one year. In the 10 cases in which estrus resumed in the first April–August after delivery in December–July, the number of days until estrus resumed was slightly shorter, with a later delivery period. On the other hand, in the three cases in which estrus resumed in May of the year after delivery, all individuals gave birth in March, suggesting that the timing of deliveries does not affect the timing of the return of estrus within one or two years. Moreover, the resumption of estrus was faster among females without offspring than for those with offspring if they gave birth in the same zoo at approximately the same time. Previous studies reported that sucking stimulation provided by offspring delayed the resumption of maternal estrus in sheep [35], goats [36], cattle (*Bos taurus*) [35,37,38], pigs [35], and Mohor gazelles (*Gazella dama mhorr*) [39]. This may also be the case for golden takins. It will be necessary to accumulate information on estrus resumption when artificial nursing is used.

## 5. Conclusions

In the present study, fecal sex steroid hormone concentration dynamics indicated that female golden takins are long-diurnal polyestrous animals. The age of sexual maturity, the start and end of the breeding season, the estrous cycle length, the relationship between sex steroid hormones and reproductive behavior, the gestation period, and the number of days of estrous regression in zoo-housed individuals were also revealed. In addition, male flehmen may be used as an external indicator to predict mating with and mounting females. The monitoring of fecal P_4_ concentrations provides important information on the estrous cycle and pregnancy, and measurements of fecal E_1_ are useful for predicting the weeks in which female golden takins will deliver.

## Figures and Tables

**Table 1:** Information on individuals and sample collection period for golden takins examined in this study.

Individual No. (Name)	Birth Date	Facility	Sample Collection Period	Age of Analysis Period	Death Date
No. 1 (Houla)	March 1990 *	TZP **	7 January 2004~26 January 2005	13.9~14.9	31 January 2005
No. 2 (Ohi)	31 March 1991 *		7 January 2004~28 February 2013	12.8~21.9	11 November 2013
No. 3 (Fuuka)	4 March 2004		2 February 2005~30 April 2019	0.9~15.2	−
No. 4 (Ouki)	20 March 2005		11 May 2005~19 April 2020	0.1~15.1	23 December 2022
No. 5 (Hoi)	10 February 2010		22 April 2013~30 April 2019	3.2~9.2	−
No. 6 (Fuku)	3 February 2017		28 September 2021~13 March 2022	4.7~5.1	−
No. 7 (Turco)	27 March 2001	YZG **	25 January 2004~2 July 2013	2.8~12.3	18 December 2014
No. 8 (Hinata)	24 February 2012		19 June 2012~19 March 2022	0.3~10.1	−
No. 9 (Mei)	7 February 2016		3 April 2018~19 March 2022	2.2~6.1	−

* Presumed date of birth for No. 1 and No. 2. ** TZP: Tama Zoological Park; YZG: Yokohama Zoological Gardens.

## Data Availability

Data are contained within the article.
